# Establishment and Characterisation by Expression Microarray of Patient-Derived Xenograft Panel of Human Pancreatic Adenocarcinoma Patients

**DOI:** 10.3390/ijms21030962

**Published:** 2020-01-31

**Authors:** Sandra Roche, Fiona O’Neill, Jean Murphy, Niall Swan, Justine Meiller, Neil T. Conlon, Justin Geoghegan, Kevin Conlon, Ray McDermott, Rozana Rahman, Sinead Toomey, Ninfa L. Straubinger, Robert M. Straubinger, Robert O’Connor, Gerard McVey, Michael Moriarty, Martin Clynes

**Affiliations:** 1 National Institute for Cellular Biotechnology, Dublin City University, Dublin 9, Ireland; 2 St. Vincent’s University Hospital, Dublin 4, Ireland; 3 Trinity College Dublin, College Green, Dublin 2, Ireland; 4 Department of Molecular Medicine, Beaumont Hospital, Royal College of Surgeons in Ireland, Dublin 9, Ireland; 5 Department of Pharmaceutical Sciences, University at Buffalo, State University of New York, Buffalo, NY 14214, USA; 6 St Luke’s Radiation Oncology Network, Dublin 6, Ireland

**Keywords:** pancreatic cancer, patient-derived xenograft, microarray

## Abstract

Pancreatic cancer remains among the most lethal cancers worldwide, with poor early detection rates and poor survival rates. Patient-derived xenograft (PDX) models have increasingly been used in preclinical and clinical research of solid cancers to fulfil unmet need. Fresh tumour samples from human pancreatic adenocarcinoma patients were implanted in severe combined immunodeficiency (SCID) mice. Samples from 78% of treatment-naïve pancreatic ductal adenocarcinoma patients grew as PDX tumours and were confirmed by histopathology. Frozen samples from F1 PDX tumours could be later successfully passaged in SCID mice to F2 PDX tumours. The human origin of the PDX was confirmed using human-specific antibodies; however, the stromal component was replaced by murine cells. Cell lines were successfully developed from three PDX tumours. RNA was extracted from eight PDX tumours and where possible, corresponding primary tumour (T) and adjacent normal tissues (N). mRNA profiles of tumour vs. F1 PDX and normal vs. tumour were compared by Affymetrix microarray analysis. Differential gene expression showed over 5000 genes changed across the N vs. T and T vs. PDX samples. Gene ontology analysis of a subset of genes demonstrated genes upregulated in normal vs. tumour vs. PDX were linked with cell cycle, cycles cell process and mitotic cell cycle. Amongst the mRNA candidates elevated in the PDX and tumour vs. normal were *SERPINB5, FERMT1, AGR2, SLC6A14* and *TOP2A*. These genes have been associated with growth, proliferation, invasion and metastasis in pancreatic cancer previously. Cumulatively, this demonstrates the applicability of PDX models and transcriptomic array to identify genes associated with growth and proliferation of pancreatic cancer.

## 1. Introduction

Pancreatic cancer remains a cancer of unmet need, with a 5 year survival rate of approximately 7−9% [1]. The survival rate has shown little to no improvement over a 30 year period. Pancreatic ductal adenocarcinoma (PDAC) has no viable screening method, and an absence of early clinical symptoms, leaving it detected usually later in the disease stage. The only curative option is surgery, and this is only viable in early stage (R0/R1) cases where the cancer has not metastasised. Even with radical surgical resection, the 5 year survival rates are not comparable with other solid cancers.

One of the most common research tools for oncology research is the cell-line model. Cell lines and cell-line panels have formed the basis of much preclinical research previously. The limitations of conventional cell lines for pancreatic cancer research has been previously discussed, showing to be a poor predictor of clinical trial outcome [2]. In pancreatic cancer, in vitro models are often poor in predicting clinical therapeutic response [3].

Patient-derived xenograft (PDX) models have emerged as a tool for investigation of pancreatic cancer and become more utilised as they have been shown to more closely represent patient tumours [4]. Due to its more aggressive nature, pancreatic cancer PDX models are established with a high take rate when compared to other cancers, such as breast cancer [5,6]. While ovarian cancer PDX models are established with similar engraftment rates as pancreatic cancer [7].

Pancreatic cancer cell lines, while not mimicking the tumour–stromal interaction or pancreatic cancer’s dense desmoplasia offer a model to examine direct drug effects on the tumour cells. PDX models also offer the opportunity for the development of novel cell-line models. The establishment of pancreatic cancer cell lines using finely minced tissue fragments and specialised media components has been previously reported [8,9]. These additional cell-line models provide valuable resources to map the genomic alterations involved in human pancreatic cancer.

This study shows the comprehensive development of patient-derived xenograft models, including detailing the tumours that failed to proliferate as PDX models. From these novel models, primary cell lines were developed and characterised as human tumour cell lines. Using microarray screening this study sought to compare the mRNA profile of adjacent normal tissue, patient tumour tissues and PDX F1 tissue. This revealed the innate heterogeneity of the adjacent normal tissue and patient pancreatic tumour but showed patient-derived xenograft models more closely clustered together. More than 1400 genes were differential expressed between the tumour and adjacent normal tissue, with more than 3800 genes differentially expressed in the tumour–PDX comparison. In this study, we focused on genes that were differentially expressed in both comparisons and associated with disease initiation and disease progression characteristics.

## 2. Results

### 2.1. Patient Characteristics

In total, 48 patient samples were collected from patients who ranged in age from 40 years to 80 years, with a total 29 males and 19 females. The baseline characteristics of the patients are given in Table 1. Of the 35 pancreatic ductal adenocarcinoma (PDAC) samples obtained, 14 (40%) were female and 21 (60%) were male. The average age for surgery for PDAC patients was 65 years for female patients and 67 years for male patients. Of the surgical specimens obtained, six samples from PDAC patients had received neo-adjuvant treatment. Node status was available for 24 of the 33 PDAC patients, with 17 of those patients having a pT3 node status. pT3 node status indicates extension beyond the pancreas into the peripancreatic soft tissue or the surrounding structures [10]. 

Due the centralised nature of pancreatic cancer surgical resection procedures in Ireland, follow-up data was not available for all patients in the study, many of whom were treated in facilities other than the surgical hospital for their post-surgical care. Available follow up information regarding treatment and survival is given in Table 2. Patients predominately received gemcitabine, gemcitabine + Abraxane or FOLFIRINOX as an adjuvant treatment, with some individuals receiving Carboplatin/Etoposide combination or Capecitabine. Patient survival ranged from less than 4 months to over 3 years.

Only 11 of 48 patient samples had recorded information regarding the patient presentation. Of those 11, nine patients presented with jaundice, obstructive jaundice or painless obstructive jaundice, 1 presented with abdominal pain and another patient was an incidental identification on a PET scan.

### 2.2. Pancreatic PDX Biobank

Tumour material obtained was confirmed macroscopically by consultant histopathologist. The quantity of tumour available for research (biobanking and implantation) was variable and determined by the pathologist. The number of mice per patient implanted varied from 3 to 5 depending on the tumour type and quantity. In total, 48 patient samples were acquired over a 5 year period. Of these, 35 samples were PDAC, with three cholangiocarcinoma, three IPMN and six NET samples acquired; a full breakdown of samples acquired and engraftment status is given in Table 3. All samples were confirmed as by the consultant histopathologist to have maintained architecture and morphology as the original patient tumour, representation images Supplementary Materials Figure S1. Of the 35 PDAC samples acquired, seven had received neo-adjuvant treatment prior to surgery, PIN 139, PIN 145, PIN 165, PIN 191, PIN 194, PIN 199 and PIN 210. Neo-adjuvant treatments included FOLFIRINOX with radiation, gemcitabine/oxaliplatin with radiation and gemcitabine/Abraxane with radiation. Of these six samples, only one sample grew as a PDX tumour. Samples were received without the complete pathological diagnostics and samples were implanted as and when they arrived. PIN 194 was a neo-adjuvant treated with gemcitabine/Abraxane and long-course radiation. On histopathological confirmation, the sample showed no trace of residual tumour cells. This suggests lower engraftment rates (14%) in pre-treated samples are possibly due to the reduced number of viable tumour cells in the sample due to the treatment effect, however a larger sample size would be required to confirm this.

**Figure 1:**
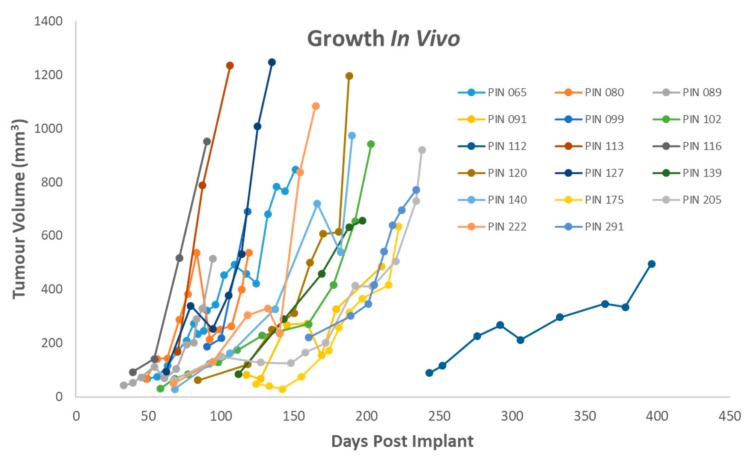
Tumour growth in vivo of patient derived xenograft (PDX) samples. PIN 099, 112, 139, 175, 190, 222, 291 represent single animals while the remainder represent an average growth rate.

The average number of days following implantation of patient tumour to palpate tumour is 78 days, ranging from 20 days to 306 days. The tumour growth rates in the first generation in mouse (F1 generation) varied both within tumours and between tumours with timelines for harvesting tumours ranging from 72 days post implant to 512 days post implant. Tumour growth rates in Figure 1 show that while tumour initiation takes time, tumour growth rate increases once established. Given that similar sized tumour pieces are implanted in each animal the intra-tumour and inter-tumour variation in growth and take rate maybe representative of innate tumour heterogeneity. In treatment-naïve PDAC samples 78% grew in vivo, in neo-adjuvant treated samples only 14% (1/7) samples produced a tumour. For neuroendocrine tumours, 20% (1/5) of samples grew in vivo.

In total, 10 F1 generation PDX fresh frozen tumour samples were investigated for mutational status by Sequenom MassArray MALDI-TOF system, Supplementary Data Table S1 and Figure S2. Of the 10 samples analysed, mutations were detected in all samples except one (PIN 116). KRAS mutation were detected in 8/10 samples and PIK3R1 mutations were detected in 5/10 samples. Other detected mutations included IDH1 (1/10); MET (1/10); PHLPP2 (3/10) and PIK3CA (1/10).

Given the small sample numbers, PDX growth and take rate cannot be attributed to a single factor but key operational factors including the immediate cooling of the sample following surgery, rapid implantation and sample selection and identification by histopathologist

### 2.3. Derivation of Cancer-Associated Fibroblasts and Tumour Cell Lines

**Figure 2:**
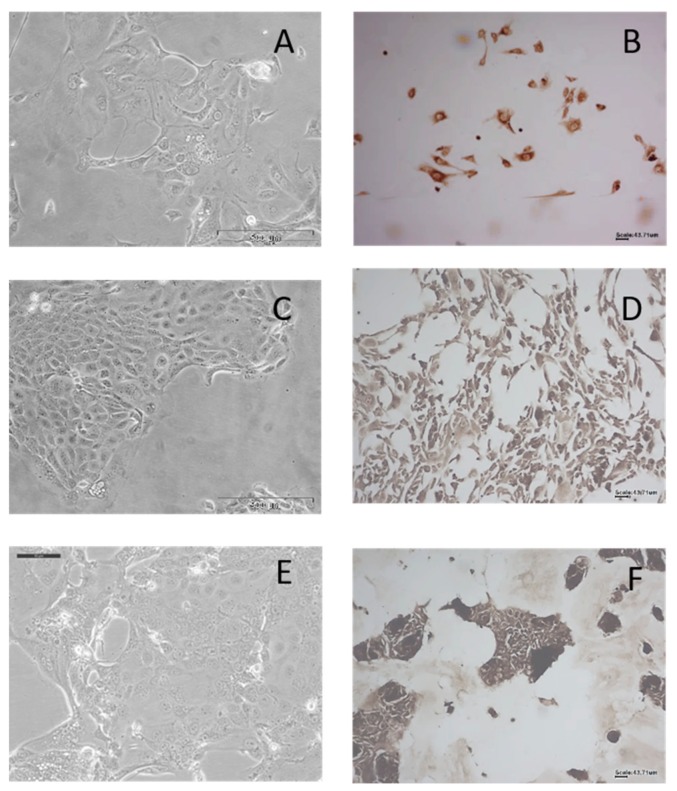
Brightfield images of cell morphology and immunohistochemical staining of PDX-derived tumour cells using primary antibody Anti-Mitochondria antibody 113–1 (1:1000 dilution) of PIN 080 (**A**,**B**) PIN 099(**C**,**D**) and PIN 127 (**E**,**F**). A, C and E were imaged at 20×, and B, D and F were imaged at 10×.

From 11 F1 PDX tumour pieces, three tumour cell lines were isolated: PIN 080, PIN 099 and PIN 127. Two further tumour cell lines failed to proliferate after initial sub-culturing: PIN 089 and PIN 091. The three tumour cell lines established were confirmed as human by IHC, as detailed in Figure 2. Immunohistochemical analysis using the human-specific antibody Anti-Mitochondria antibody 113–1 (dilution 1/1000) confirmed that the tumour cells isolated were human in origin.

From seven patient tumours, two patient-derived stromal cell lines were isolated. The two stromal cell lines, Fibro-102 and Fibro 120, have been successfully cultured, cryo-preserved and restored. These cell lines grow with a fibroblast morphology. Preliminary data (unpublished) indicated phenotypic changes in pancreatic cancer cells (Mia-PaCa-2, Panc-1) in response to co-culture with patient-derived stromal cells in comparison to cancer cell alone.

### 2.4. Immunohistochemical Characterisation of PDX tumours

**Figure 3:**
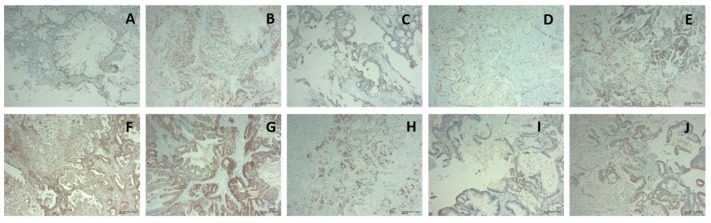
Immunohistochemical staining of PDX tumour sections using primary antibody Anti-Mitochondria antibody 113-1, where panel **A** shows PIN 065; **B** PIN 080; **C** PIN 089; **D** PIN091; **E** PIN 099; **F** PIN 120; **G** PIN 140; **H** PIN 141; **I** PIN 160; **J** PIN 161. Magnification for all images is 10× (scale 43.71 µm) and an antibody dilution of 1/1000.

PDX tumours are known to recapitulate the patient morphology. Additionally, as the tumours grow, within the first passage in the stroma is replaced with mouse tissue [2,11,12]. Shown in Figure 3 are representative examples of 10 PDX tumour samples stained for human specificity using Anti-Mitochondria antibody 113-1 (1/1000 dilution). The images show the tumour cells staining positive for human with the stromal material not staining. This demonstrates the maintenance of the human tumour phenotype through the passaging, with murine cells overtaking the stroma. 

### 2.5. Microarray Gene Expression Analysis

**Figure 4:**
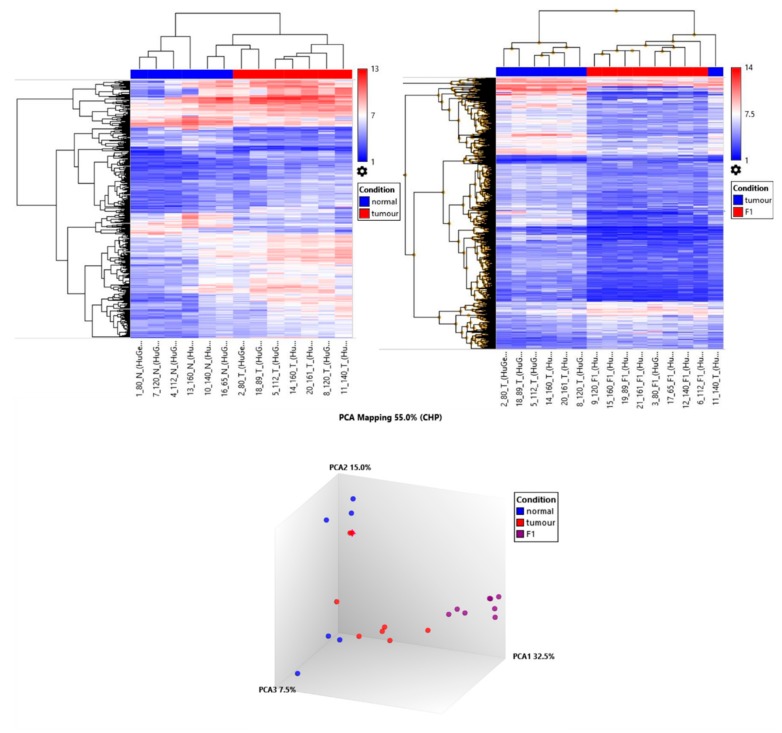
Hierarchical clustering and principle component analysis of adjacent normal tissue, patient tumour and PDX tumour.

Microarray gene expression profiling was performed on GeneChip Human Gene 2.0 ST array on five triple-matched samples and three double-matched samples, detailed in Table 4. mRNA profile showed that after passaging in the mouse, the PDX tumours cluster closely together. The primary patient tumours predominantly cluster together by PCA analysis, with two samples PIN 080 and PIN 089 distant from the cluster, Figure 4. The adjacent normal tissue displayed the least amount of clustering. This is representative of the heterogeneity of the patients with the minor selection pressure of the PDX formation and the murine stromal replacement through engraftment resulting in a less heterogeneous sample population in the PDX tumours.

In total, more than 5000 genes were differentially expressed in the comparisons on adjacent normal vs. tumour and tumour vs. PDX, taking a 1.5-fold cut off. The breakdown of the numbers of genes changed is given in Table 5, while the top 20 genes differential expressed with a LOG fold change of 2 or greater in the adjacent normal vs. tumour and tumour vs. PDX F1 is shown in Table 6; Table 7, respectively.

In order to determine which biological process the differentially expressed genes were involved in, gene ontology analysis was completed on a short list of 89 genes [13,14]. Gene ontology (GO) analysis showed that the shortlist of 89 genes upregulated by two-fold or more in both the N vs. T comparison and T vs. F1 comparison were predominantly related to biological processes such as cell cycle and mitosis (Table 8). This suggests that this shortlist of genes (Supplementary Table S2) are selected for growth and demonstrate the genes associated with tumour initiation and tumour progression. Further validation was performed using AMIGO database against Homo Sapiens database of all genes. [15]. The shortlisted 89 genes of interest were classified by biological process using a Fisher Test and applying a Bonferroni correction (Table 9). The complete dataset in this publication have been deposited in NCBI’s Gene Expression Omnibus, GEO Series accession number GSE141873 [13,14,16].

**Figure 5:**
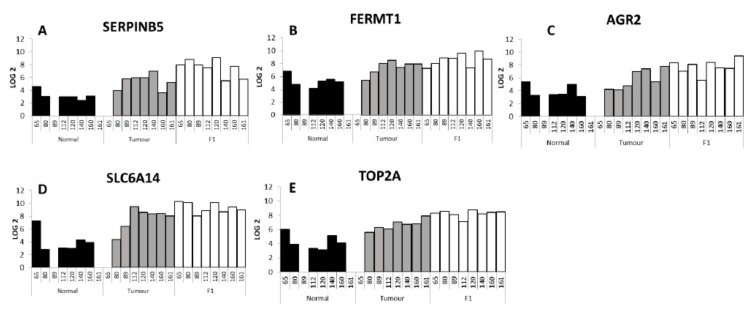
Individual expression of five selected genes, *SERPINB5* (**A**) *FERMT1* (**B**) *AGR2* (**C**) *SLC6A14* (**D**) *TOP2A* (**E**), across the adjacent normal, patient tumour and PDX F1 samples. PIN 089 and PIN 161 adjacent normal samples were not included in the microarray analysis, and PIN 065 patient tumour sample.

Figure 5A–E show five genes of interest that were upregulated in N vs. T vs. F1 giving the individual expression in each sample. In our global analysis of six adjacent normal compared to seven tumour samples, SERPINB5, also known as P15 and Maspin [17], was shown to be increased in the tumour samples compared to the adjacent normal samples (6.8-fold increase), with a further increase in the F1 generation PDX tumours (eight samples) compared to the patient tumour samples (6.6-fold increase). Both of these global comparisons were statistically significant (*p* = 8.84 × 10^-5^ and 8.96 × 10^-6^ respectively). Figure 5A shows the individual levels of SERPINB5 in the adjacent normal, tumour and F1 PDX tumour samples. In the comparison of FERMT1 (Figure 5B) compared to patient tumour a 6.4-fold increase (*p* = 3.00 × 10^-4^) was detected with a further 2.5-fold increase (*p* = 5.40 × 10^-3^) in the F1 PDX tumour material when compared to the F1 cohort. AGR-2 (Figure 5C) was increased 4.98-fold (*p* = 1.50× 10^-3^) in the patient tumour compared to the adjacent normal tissue and a further 5.09-fold (*p* = 3.00 × 10^-4^) increased in PDX F1 tissue compared to tumour material Solute Carrier SLC6A14 (Figure 5D) showed a 22.8-fold increase (*p* = 4.00 × 10^-4^) in a comparison between tumour tissue and adjacent normal material and a further 3.2-fold increase (*p* = 1.07 × 10^-2^) in the F1 PDX cohort compared to the tumour tissue. TOP2A (Figure 5E) showed a 6.4-fold increase (*p* = 2.00 × 10^-4^) in the tumour compared to adjacent normal tissue (*p* = 2.00 × 10^-4^) and a further 3.8-fold increase in the PDX F1 tissue compared to the patient tumour. MUC1 has been shown to be overexpressed in pancreatic cancer [18], and has been associated with multidrug resistance and gemcitabine resistance [19]. In our list of differentially expressed genes MUC1 was 1.7-fold increased in tumour vs. normal but not statistically significantly changed in the tumour compared to F1 comparison (see Supplementary Table S2). MUC13 was significantly increased in both the N vs. T comparison (9.4-fold) and the T vs. F1 comparison (3.0-fold). MUC13 has been shown to be increased in PDAC tissue compared to normal adjacent tissue [20].

## 3. Materials and Methods

### 3.1. Sample Acquisition and Ethical Approval

Pancreatic cancer tissue and adjacent normal tissue (N) were obtained from patients undergoing surgical resection at St Vincent’s University Hospital. After initial macroscopic pathological confirmation, material remaining after diagnostic sampling was cold transferred in RPMI 1640 medium containing 1% Penicillin-Streptomycin, 1% fungazone to DCU. Transfer time between hospital and implantation was on average 2 h or less.

Collection of patient material was approved by St Vincent’s University Hospital Research Ethics Committee. All animal work received ethical approval from the DCU Research Ethics Committee (DCUREC/2012/202) and was licensed by Department of Health (B100-4501).

### 3.2. PDX Tumour Development

The tumour was cut into implant-sized pieces (<2 mm^3^) and rinsed with fresh serum-free RPMI media following transport. Severe combined immunodeficiency (SCID), CB17/lcr-Prkdc^scid^/lcrCrl mice (Charles River, UK) were implanted subcutaneously with fresh patient tumour material. Depending upon the size and type of tumour material, 3–5 mice were implanted per patient sample. Under anaesthesia (isoflourane, O_2_ carrier gas) a small incision was made in the skin of the left flank of the animal. The tumour piece was placed in the pocket under the skin and the wound sealed with a single staple. The animals were monitored post-surgery, and staple removal was within 10 days. Animals were monitored weekly for body weight and tumour development. Mice were monitored for tumours development for up to 1 year post implantation. Animal welfare monitoring criteria included tumour volume, tumour axis, body weight and condition. There tumour volume and tumour axis limits were set as <2000 mm^3^, and <20 mm respectively. A decrease in body weight of >10% resulted in increased monitoring with body weight decrease of 20% resulting in humane euthanasia.

### 3.3. Preservation of PDX tumours

Following humane euthanasia of the mouse, the tumour was divided for cryopreservation, formalin-fixed paraffin embedding (FFPE) and snap frozen. For cryopreservation, implant-sized tumour pieces were stored in freezing media (RMPI/FCS/DMSO—50/40/10% v/v) in a cryovial and placed in a Mr Frosty^TM^ freezing container (ThermoFischer, Dublin Ireland) for 24 h and then transferred to the liquid nitrogen (LN_2_) for long-term storage. For snap freezing, a piece of tumour was minced with a scalpel, placed in a cryovial and placed directly into LN_2_, then stored at −80 °C. For FFPE, a central slice was preserved in 10% neutral buffered formalin overnight and processed subsequently in 70%, 90%, 100% ethanol followed 100% xylene (1 h each). Following processing the tumours were embedded in paraffin. FFPE samples of PDX tumours were reviewed by consultant histopathologist to validate maintenance of tumour phenotype in comparison with original patient tumour.

### 3.4. Cell Isolation from Tumour Material

A small piece of tumour (<2 mm^3^) was minced and placed in Collagenase/Hyaluronidase (StemCell Technologies Cambridge, UK) at 4 °C for 1 h and then transferred to 37 °C for 30 min. Following digestion, the sample was centrifuged 200 g for 5 min and the supernatant removed. The digested material was suspended with complete media (DMEM/F-12 Hams supplemented with 10% FCS 1% PenStrep). Samples were monitored for growth and fed as required. Tumour cells were isolated from first generation PDX tumours while fibroblasts were isolated directly from patient tumours. Tumour cell lines were confirmed as human by IHC.

### 3.5. Immunohistochemical Analysis of PDX tumours to determine Human-Mouse Tissue

#### 3.5.1. Immunohistochemistry

All immune-histochemical (IHC) staining was performed using the DAKO Autostainer. Deparaffinisation and antigen retrieval were performed using Epitope Retrieval 3-in-1 solution (pH 6) (DAKO, Cruinn, Dublin Ireland) was used. For epitope retrieval slides were heated to 97 °C for 20 min and then cooled to 65 °C.

#### 3.5.2. Real Envision Detection System Peroxidase/DAB+

Following either deparaffinisation or epitope retrieval as outlined above slides were immersed in 1× wash buffer. On the autostainer (DAKO), slides were blocked for 10 min with 200 µL HRP Block (DAKO). Slides were washed with 1× wash buffer and 200 µL of the primary antibody were added to the slides for 30 min. The primary antibody used was Abcam Anti-Mitochondria antibody 113–1 (ab92824), a human-specific antibody [21]. The antibody was used at a dilution of 1 in 1000. Slides were once again washed with 1× wash buffer and then incubated with 200 µL Real EnVision (DAKO) for 30 min. Slides were washed again with wash buffer and then stained with 200 µL DAB+ substrate for 5 min and this procedure was repeated twice. All slides were then counterstained with haematoxylin (DAKO) for 5 min and were rinsed with deionised water and then with wash buffer. A negative control sample was also tested for each sample using antibody diluent without any antibody present. This was used to evaluate any non-specific staining. Following the counterstaining with haematoxylin, the slides were then dehydrated. This was achieved by immersing the slide in 70%, 90% and 100% ethanol, twice in each ethanol solutions for 3 min. The slides were then immersed into xylene, twice, for 5 min each. Once the slides were cleared, they were mounted using DPX (BDH).

#### 3.5.3. Immunohistochemical Analysis of Cell Lines

Cells were seeded directly on Superfrost^®^ microscope slides and allowed to attach overnight at 37 °C, 5% CO_2_. Slides were washed 3 times in PBS and cells were then fixed in cold 4% paraformaldehyde for 15 min and washed with PBS. Immunostaining was carried out using the Dako Autostainer as per Section 3.5.2, without the initial antigen retrieval.

### 3.6. Mutational Analysis

Mass spectrometry-based single-nucleotide polymorphism genotyping technology (Agena Biosciences, Hamburg, Germany) was used for identification of hotspot, potentially clinically relevant nonsynonymous somatic mutations as previously described [22]. The genes were further subdivided by pathway, and include MAPK, PI3K and related pathway genes (Supplementary Table S3). Assays were designed using strict assay design parameters optimized for sensitive mutation detection. The panel consisted of 31 multiplex assays capable of detecting 378 somatic hotspot mutations in 49 genes. DNA was extracted from fresh frozen tumour material using QIAmp DNA mini kit (Qiagen, Hilden, Germany) and quantified by Qubit dsDNA. 10 ng of DNA was added to each PCR reaction and DNA was amplified using custom designed PCR primer pools. Unincorporated nucleotides were inactivated using shrimp alkaline phosphatase (SAP), and a single base extension reaction was performed using extension primers that hybridise immediately adjacent to the mutations of interest. Salts were removed by adding a cation-exchange resin, before the multiplexed reactions were spotted onto SpectroCHIP II arrays. Matrix chips were analysed on an Agena MassArray Matrix-assisted laser desorption/ionisation Time of Flight (MALDI-TOF) system.

### 3.7. Sample Preparation for Microarray

Snap-frozen sections of PDX tumours, original patient material and adjacent normal tissue were prepared for RNA extraction by grinding samples under LN_2_ using a mortar and pestle. Briefly, tissue sample was placed in a metal mortar with a small volume of LN_2_ and ground quickly. When powdered the powder was transferred to a clean Eppendorf tube. This was done before the LN_2_ completely evaporated and while the powder was still frozen.

Once homogenised, RNA samples were prepared using Trizol, according to manufacturer’s guidelines. Briefly, powdered tissue was lysed in 1 mL of Trizol reagent. Sample was allowed to homogenise at room temperature for 15 min. Per 1 mL of Trizol reagent used, 200 µL of chloroform was added to the Eppendorf (Hamburg, Germany), sample was mixed and allowed to sit for 5 min. At 4 °C samples were spun at 12,000× *g* for 15 min. The aqueous phase was then separated using a micropipette and places in a new Eppendorf. 500 µL of isopropanol was added to the aqueous phase, allowed to sit at room temperature for 10 min, after which it was spun at 12,000× *g* at 4 °C for 10 min. The RNA pellet was then washed using 75% ethanol and spun at 4 °C at 7500× *g* for 5 min. Ethanol was removed from pellet, which were allowed to air dry, and were then reconstituted in nuclease free water.

Samples were quantified using a Nanodrop (Thermo Scientific, Dublin Ireland) and quality determined using Agilent Bioanalyser. Samples with a RNA Integrity Number (RIN) number greater than 8 were most suitable for microarray analysis.

### 3.8. Microarray GeneChip Human Gene 2.0 ST array Processing and Hybridization

Preparation of cRNA, hybridization, and scanning of microarrays was performed according to the manufacturer’s protocol (Affymetrix, Thermo-Fischer, Santa Clara, Ca, USA). In brief, 300 ng of total RNA extracted from homogenised patient samples isolated using Trizol was converted into double-stranded cDNA by reverse transcription. Biotin-labelled cRNA was generated by converting the cDNA sample using the Genechip WT plus reagent kit (Affymetrix, Thermo-Fischer, Santa Clara, Ca, USA). Labelled cRNA was hybridized to the Affymetrix GeneChip^®^ Human Gene 2.0 ST Array while rotating at 60 rpm for 16 h at 45 °C. After hybridization, the microarray was washed using the Affymetrix Fluidics Station according to the manufacturer’s protocol. The chips were scanned in an Affymetrix 3000 7G scanner.

Differential gene analysis expression was carried out using Applied Biosystems Transcriptome Analysis Console (TAC) software 4.0.2. Resulting gene lists were filtered for +/- 2-fold changes, a *p*-value < 0.05 and an overall FDR F-Test: <0.005.

## 4. Discussion

Patients ages and diagnosis characteristics are broadly representative of the earlier-stage pancreatic cancer patient population at large. In Ireland, the median age of diagnosis is 70–74 years for the entire pancreatic patient population [23]. In this study, the average age of 65 years for women and 67 years for men shows that patients who are eligible for surgery are somewhat younger than the pancreatic patient population as a whole. Only 10–20% of patients diagnosed with pancreatic cancer are eligible for surgery, and the biobank developed here is representative of that patient population [24,25]. The ability of a patient tumour to grow a tumour in vivo was not correlated with any single characteristic, though PDAC samples, which formed the bulk of the samples received, formed tumours at a greater rate than neuroendocrine tumours (78% compared to 20%).

PDAC samples which were treatment naïve formed tumours in vivo 78% of the time. This is somewhat higher that previously reported rates of 61% (sub-cutaneous) and 62% (orthotopic) for primary pancreatic cancer samples, though these models were established in nude mice rather than SCID mice [26,27]. The 78% reported here is also higher than 43% detailed by Pergolini et al., though Pergolini et al. reported cryopreserving tumours first rather than direct implantation which potential reflects the differences seen here [28]. This suggests that for initial passaging, direct implantation gives a better take rate with cryopreservation being suitable for later passages. This is potentially due to the selection pressures the tumour undergoes in vivo and the biological processes involved in engraftment.

From seven tumours collected where the patient had received neo-adjuvant treatment, one formed a sub-cutaneous tumour in vivo. Dorado et al. examined three experimental techniques for the establishment of pancreatic PDX models, subcutaneous, orthotopic and intraperitoneal. Of the bank of 11 samples collected in that study, one had received neo-adjuvant therapy and formed a sub-cutaneous tumour, but did not form a tumour intraperitoneal nor at the orthotopic site [29].

Previously, our group examined 10 primary patient tumour samples and nine matched normal adjacent specimens and 10 matched F1 and F2 generation PDX tumours using high-resolution mass spectrometry. MS identification allowed for the isolation of the human only protein, representing the tumour cells, and these were tracked from patient tumour, to F1 and F2 generation PDX tumours. Between patient primary and F1 tumour 32 proteins were upregulated and 113 downregulated. In comparison, between F1 and F2 generation of PDX tumour, only eight human-specific proteins were differentially expressed when analysed by quantitative label-free differential analysis. This demonstrates the fidelity of tumour phenotype once engrafted [30]. This is in line with previously published data the shows, once established, xenografts tend to be robust with stable gene expression profile between early and late passage PDX tumours [31]. This bank of pancreatic PDX tumours models the subset of pancreatic cancer patients who are eligible for surgical resection. Mutational analyses by Sequenom MassArray MALDI-TOF showed 80% of samples examined possessed a KRAS mutation. This is in line with previously reported clinical KRAS mutation rates [32]. These PDX samples are a truer representation of human pancreatic cancer than pancreatic cancer cell lines or cell-line derived xenograft models, which often fail to recapitulate the stroma and desmoplasia of pancreatic cancer. In recent years, given the unmet need of pancreatic cancer patients, banks of patient-derived xenograft tumours have been established. The bank established in this study displays a higher engraftment rate for PDAC tumours than many others previously reported. Two recent reports showed similar engraftment rates (72% and 71%), although this was observed in more immune compromised NSG and NSG nude mice [33,34].

Eight PDX tumour samples were interrogated by Affymetrix Microarray technology in matched comparison to patient tumour and adjacent normal tissue. Primary patient tumour cluster together with PDX tumours clustering together but separate from the primary tumour. RNASeq data in ovarian PDX models demonstrate this differential clustering is predominantly due to the loss of human stroma and the growth of murine stroma [35]. By comparing the adjacent normal to the tumour and the tumour to the F1 PDX tumour we aimed to map the genes associated with tumour growth, proliferation and tumour in engraftment. Gene ontology analysis of the 89 gene increased in tumour (vs. adjacent normal) and further increased in PDX tumour (vs. patient tumour) showed the biological processed enriched for genes associated with cell cycle, cell cycle process as well as mitotic cell cycle processes (Table 8 and Table 9). A selection of the genes shortlisted and described here, have previously been associated with pancreatic cancer and validated as having an effect on proliferation, invasion and migration.

Serpin family B member 5, *SERPINB5*, had the largest fold change in the T vs. F1 analysis and in the top ten differentially expressed genes in the T vs. N analysis. The SERPIN superfamily of proteins is known to consist of 37 members in humans, with 13 members of the SERPINA family and 13 members of the SERPINB family [36]. SERPINA3 has been associated with endometrial cancers [37] and undifferentiated carcinoma with osteoclast-like giant cells, a rare pancreatic cancer [38]. In our differential gene expression list 3 of the 39 SERPIN family members were statistically significantly changed, *SERPINA1, SERPINB5* and *SERPINE1*. *SERPINA1* was increased in T vs. N (3.6-fold), but decreased in T vs. F1 (5.0-fold). *SERPINE1* was decreased in T vs. N analysis (3.6-fold) and further decreased in T vs. F1 comparison (4.9-fold). *SERPINB5* has been previously associated with pancreatic cancer. In a meta-analysis of publicly available datasets of transcriptome data for human pancreas specimens and a GEM mouse models Bhasin et al. included *SERPINB5* as one the of the genes in a five gene panel for discriminating PDAC and early precursor lesions. Other genes included in this panel were *TMPRSS4, AHNAK2, POSTN, ECT2* [39]. The other four genes in the panel were also detected as being significantly changed in our analysis. *TMPRSS4* showed fold changes of 9.5 and 2.1 in the N vs. T and T vs. F1 respectively. Similarly, *AHNAK2* fold changes were 2.6 (N vs. T) and 2 (T vs. F1), *POSTN* 15.5 (N vs. T) and -69.9 (T vs. F1), *ECT2* 2.6 (N vs. T) and 2.7 (T vs. F1). RNA-Seq analysis by Mao et al. of 10 PDAC tumour samples and adjacent benign tissue also detected *SERPINB5*. Their analysis showed *SERPINB5* to be overexpressed in PDAC tumour in comparison to adjacent normal tissue [40]. Also in 2010, Mardin et al. identified through the analysis of the invasive and metastatic potential of 16 PDAC cell lines that *SERPINB5* expression may correspond with invasive tumours [41]. Previous proteomic analysis of the same sample cohort also found *SERPINB5* to be increased at the protein level when human-specific proteins were identified with significantly increased expression in PDX F1 tumours compared to PDAC tumour tissues, as determined by quantitative label-free mass spectrometric analysis [30].

Fermitin family member 1, *FERMT1*, encoded protein is involved in integrin signalling and linkage of the actin cytoskeleton to the extracellular matrix and has been shown to be significantly over-expressed in colon cancer [42]. FERMT1 has been shown to interact directly with β-catenin and activated the Wnt/β-catenin signalling pathway by decreasing the phosphorylation level of β-catenin in colon cancer. This activation was seen to promote EMT and led to a more aggressive and invasive phenotype in colon cancer [43]. In oesophageal cancer, overexpression of *FERMT1* by lenti-viral vector increased proliferation and radiation resistance in vitro [44]. A study by Fukuhisa et al. examined the role of EPS8 in PDAC, and showed that EPS8 was overexpressed in PDAC clinical specimens, and linked with proliferation and invasion in vitro. *FERMT1* is a putative downstream gene target of EPS8 [45]. While changes in *EPS8* were not seen in our data set, *FERMT1* changes were seen. To date, *FERMT1* changes have not previously been directly shown in pancreatic cancer.

Ramachandran et al. showed that Anterior Gradient 2, *AGR2*, gene was expressed in pancreatic cancer tissues and cell lines, though this is not expressed in chronic pancreatitis samples. In vitro, *AGR2* silencing by siRNA knockdown showed a decrease in cancer cell proliferation and invasion and increased gemcitabine sensitivity of resistant cells [46]. In breast cancer patients, *AGR2* was associated with poorer survival and increased metastasis [47]. In pancreatic cancer, the invasive potential of pancreatic cancer cells in vitro was proportional to the *AGR2* expression level [48].

Solute carrier family 6 member, SLC6A14 encoded protein is a sodium- and chloride-dependent neutral and basic amino acid transporter [49]. In breast cancer mouse models, *SLC6A14* knock out mice showed a marked delay in tumour formation and tumour size, and analysis of the tumour material revealed evidence of amino acid starvation in the slc6a14^-/-^ mice suggesting a key role from SLC6A14 in both tumour development and growth [50]. In 2019, Cheng et al. used three publicly available datasets to identified differentially regulated genes and validated these genes in a retrospective clinical study. This highlighted *SLC6A14* as a potential prognostic biomarker of pancreatic cancer. Interestingly *CENPF* and *SERBINB5* were also included in this panel of prognostic genes. Both of these genes were also identified in our panel [51]. SLC6A14 has been investigated as a potential novel therapeutic target for pancreatic cancer using α-methyltryptophan as a pharmacological inhibitor of SLC6A14 and showed reduced proliferation and clonogenic survival [52].

Topoisomerase IIα (*TOP2A*) gene encodes for a nuclear enzyme that regulates the topological structure of DNA and is involved in chromosome condensation, chromatid separation and torsional stress relief during DNA transcription and replication. TOP2A has been associated with many solid cancers such as breast and prostate cancers [53,54,55]. However, in a study of 24,262 patients with diverse tumour types, only 4% of tumours had *TOP2A* amplification [56]. Pancreatic patients with higher TOP2A levels were thought to have poor prognosis. TOP2A has been shown in pancreatic cancer cell lines to be associated with proliferation and migration through activation of the β-catenin signalling pathway [57].

## 5. Conclusions

This study details the establishment of a PDX biobank containing pancreatic ductal adenocarcinoma, intraductal papillary mucinous neoplasm, cholangiocarcinomas and neuroendocrine tumour including one PDX of a neo-adjuvant treated PDAC model. From this biobank, a further of three primary tumour cell lines and two primary patient-derived stromal cell lines were established.

This panel of PDX tumours with matched patient tumour and adjacent normal tissue was investigated by Microarray. A subset panel of 89 genes were identified and shortlisted as differentially upregulated in tumour compared to normal and further upregulated in PDX F1 tumours. Potentially this panel indicates genes highly associated with tumour growth and development. These genes were predominantly associated with proliferation, cell cycles and mitotic processes, suggesting a significant role in tumour proliferation, progression and tumour engraftment in vivo.

## Figures and Tables

**Table 1:** Patient diagnostic information.

Patient ID	Diagnosis	Age at Surgery (Years)	Sex	Surgical Procedure	Grade	Resection Status	Location	Size (mm)	Background Pathology
PIN 062	Islet cell carcinoma (ICC)	66.1	M	Whipple’s	Moderately differentiated	-	-	15	-
PIN 065	Invasive ductal adenocarcinoma (PDAC)	77.4	F	Whipple’s	Moderately differentiated	-	-	50	-
PIN 080	Invasive adenocarcinoma	70.6	M	Whipple’s	Moderately differentiated	-	-	8	-
PIN 082	Intraductal papillary mucinous neoplasm (IPMN)	63.8	M	Total Pancreatectomy Incl Spleen	Low- and high-grade dysplasia	-	-	60	-
PIN 089	Invasive ductal adenocarcinoma	45.9	F	Distal Pancreas	Moderately differentiated	-	-	35	-
PIN 091	Invasive ductal adenocarcinoma	64.5	M	Median Pancreatectomy	Moderately invasive	-	-	21	PanIN 2 and focal chronic pancreatitis
PIN 099	Invasive ductal adenocarcinoma	63.0	F	Whipple’s	Poorly differentiated	R0	Pancreatic Head	17	Pancreatic intraepithelial neoplasia, low-grade (PanIN1) and marked chronic pancreatitis
PIN 102	Cholangiocarcinoma	61.8	M	Whipple’s	Poorly differentiated	-	-	23	-
PIN 112	Invasive ductal adenocarcinoma	79.7	M	Distal Pancreas	Moderate differentiation	-	-	20	Chronic pancreatitis
PIN 113	Poorly differentiated adenocarcinoma with anaplastic and signet ring	74.7	M	Distal Pancreas	Poorly differentiated	-	-	145	-
PIN 115	Invasive ductal adenocarcinoma, moderately differentiated	72.8	F	Distal Pancreatectomy	Moderately differentiated	-	-	35	Chronic pancreatitis and PanIN of the main pancreatic duct
PIN 116	Invasive ductal carcinoma	62.4	M	Distal Pancreas	Poorly differentiated	-	Pancreatic Tail	60	Prominent associated chronic pancreatitis
PIN 120	Invasive ductal adenocarcinoma	65.1	M	Whipple’s	Moderately differentiated	-	Head of Pancreas	14	The pancreatic parenchyma shows PanIN3 and focal chronic pancreatitis
PIN 127	Invasive moderately differentiated adenocarcinoma	72.7	M	Whipple’s	Moderately differentiated	-	-	48	PanIN Ib is noted in the pancreas
PIN 132	Metastatic renal cell carcinoma (RCC)	71.2	M	Whipple’s	-	-	-	25	-
PIN 135	Neuroendocrine tumour (NET)	70.9	M	-	-	-	-	13	-
PIN 137	Invasive ductal adenocarcinoma	68.9	M	Whipple’s	Moderately differentiated		Uncinate Process	30	Chronic pancreatitis
PIN 138	Invasive adenocarcinoma, intestinal type	60.1	M	Whipple’s	Moderately differentiated	R0	-	19	Stomach with chronic gastritis, mild activity and focal chronic pancreatitis
PIN 139	Invasive ductal adenocarcinoma with squamous differentiation	70.8	M	Pancreaticoduodenectomy (Whipple’s resection)	Poorly differentiated	-	Uncinate Process	30	-
PIN 140	Invasive ductal carcinoma	63.5	M	Pancreaticoduodenectomy, Whipple’s procedure	Moderate	R0	Pancreatic Head	32	Invasive tumour arises in association with an IPMN which displays intestinal differentiation and contains moderate dysplasia. The invasive tumour present is pancreatico-biliary type (non-mucinous). Focal chronic pancreatitis and chronic inactive gastritis also noted
PIN 141	Invasive ductal adenocarcinoma	82.7	M	Whipple’s	Poorly differentiated	-	Pancreatic Head	35	Chronic pancreatitis
PIN 145	Post chemoradiotherapy - tumour presumed necrotic	71.2	M	Whipple’s	-	-	-	32 (50 mm on pre-neoadj CT)	-
PIN 148	invasive ductal adenocarcinoma	62.9	F	Distal Pancreatectomy	Poorly differentiated	-	-	15	-
PIN 160	Invasive ductal adenocarcinoma, moderately differentiated	77.4	F	Whipple’s	Moderately differentiated	-	-	25	Extensive associated chronic pancreatitis
PIN 161	Invasive ductal adenocarcinoma	61.3	M	Whipple’s	Moderately differentiated	-	Uncinate Process/Pancreatic Head		High-grade PanIN
PIN 165	Invasive ductal carcinoma	57.4	F	Whipple’s	Poorly differentiated	-	Pancreatic Head and Uncinate Process	35	-
PIN 175	IPMN/NET	63.1	M	Whipple’s	-	-	-	27	-
PIN 190	Invasive ductal adenocarcinoma	71.8	M	Whipple’s	Poorly differentiated	-	Pancreatic Head	25	-
PIN 191	Ductal adenocarcinoma post neo-adjuvant chemotherapy	70.3	F	Central and Distal Pancreas	Moderately differentiated, entrapped within a densely fibrotic stroma showing chronic inflammation and evidence of treatment effect	-	-	-	-
PIN 194	No evidence of residual tumour—complete pathology response	71.6	F	Whipple’s	-	-	-	0	-
PIN 199	Invasive ductal adenocarcinoma neo-adjuvant chemotherapy and radiotherapy	62.2	M	Distal Pancreatectomy with Splenectomy	Moderately differentiated	-	-	15	PanIN grade 3
PIN 205	Invasive ductal carcinoma	63.6	F	Pancreatic Duodenectomy (Whipple’s Resection)	Moderately differentiated	R1	Pancreatic Head	20	PanIN, chronic pancreatitis
PIN 210	Well-differentiated neuroendocrine tumour (NET)	65.4	M	Whipple’s Resection) Pancreaticoduodenectomy	G1 (1 mitoses/10 hpf, Ki67 index = 5%)	-	-	-	-
PIN 211	Invasive ductal adenocarcinoma	80.0	M	Whipple’s/Pancreatico-duodenectomy:	Well differentiated	R1	Pancreatic Head	25	Chronic pancreatitis Pancreatic divisum
PIN 212	Mucinous cystic neoplasm	63.4	F	Distal Pancreas and Spleen	-	-	-	100	-
PIN 213	Invasive adenocarcinoma/cholangiocarcinoma	70.6	F	Pancreatico-duodenectomy: Whipple’s	Moderately differentiated	R0	Distal Common Bile Duct (Intra-Pancreatic)	20	PanIN 1b: Chronic pancreatitis and chronic cholecystitis,
PIN 214	Well-differentiated neuroendocrine tumour	79.2	F	Distal Pancreatectomy with Spleen	G2 (3 mitoses/10 hpf, Ki-67 > 5–20%)	R0	-	60	-
PIN 218	Well-differentiated neuroendocrine tumour (NET)	47.7	F	Pancreaticoduodenectomy (Whipple’s Resection)	G1 (<2 mitoses/10 hpf).	R1	Head and Neck of Pancreas	-	-
PIN 222	Invasive ductal carcinoma	64.5	M	Pancreaticoduodenectomy (Whipple’s Resection)	Moderately differentiated	R0	Pancreatic Head		Chronic pancreatitis, chronic cholecystitis and cholelithiasis
PIN 226	Invasive ductal adenocarcinoma	71.6	F	Pancreaticoduodenectomy (Whipple’s Resection)	Moderately differentiated	R0	-	40	-
PIN 228	Invasive ductal adenocarcinoma	77.1	M	Pancreaticoduodenectomy (Whipple’s Resection)	Moderately differentiated	R0	-	-	-
PIN 233	Invasive ductal adenocarcinoma	73.0	F	Pancreaticoduodenectomy (Whipple’s Resection)	Moderately differentiated	R0	Pancreatic Head	-	-
PIN 238	Neuroendocrine tumour	40.7	M	Whipple’s	-	-	-	-	-
PIN 239	GIST*	57.6	F	-	-	-	-	-	-
PIN 266	Cholangiocarcinoma	57.0	F	Whipple’s	-	-	-	-	-
PIN 268	Invasive ductal adenocarcinoma	57.3	M	Whipple’s	Poorly differentiated	R1	-	-	Chronic Pancreatitis
PIN 277	Adenocarcinoma	69.2	M	Pancreaticoduodenectomy (Whipple’s Resection)	-	R0	-	-	PanIN Grade 1
PIN 291	Adenocarcinoma, pancreaticobiliary type	56.5	F	Pancreaticoduodenectomy (Whipple’s Resection)	-	R1	-	-	-

* GIST = Gastro-intestinal stromal tumour; “-“ = information not available

**Table 2:** Patient survival and treatment information.

Patient ID	Diagnosis	Time to Last F/U (Years)	Status at Last F/U	Neo Adjuvant Treatment	Adjuvant Treatment
PIN 062	ICC	3.7	Alive	None	Gem
PIN 065	PDAC	1.0	Deceased	None	Adj radiation, no adj chemo
PIN 080	PDAC	0.8	Alive	None	Gem
PIN 082	IPMN	3.6	Alive	None	No adj chemo
PIN 089	PDAC	1.6	Alive	None	No information
PIN 091	PDAC	3.1	Deceased	None	Gem/Oxaliplatin + radiation
PIN 099	PDAC	1.6	Deceased	None	Gem
PIN 102	Cholangio	1.7	Deceased	None	Adjuvant treatment—regime unknown
PIN 112	PDAC	2.6	Alive	None	Gem
PIN 113	PDAC	0.4	Deceased	None	Gem
PIN 115	PDAC	0.0	Alive	None	-
PIN 116	PDAC	1.1	Deceased	None	Gem/Abraxane
PIN 120	PDAC	1.1	Alive	None	Gem
PIN 127	PDAC	2.1	Deceased	None	Capecitabine
PIN 132	RCC	2.4	Alive	None	-
PIN 135	NET	1.6	Alive	None	-
PIN 137	PDAC	2.4	Alive	None	Gem/Abraxane*
PIN 138	PDAC	1.7	Alive	None	Adjuvant treatment + radiation
PIN 139	PDAC	1.4	Deceased	FOLFIRINOX + Radiation	Adjuvant treatment—regime unknown
PIN 140	PDAC	0.4	Deceased	None	Adjuvant treatment—regime unknown
PIN 141	PDAC	1.1	Deceased	None	No information
PIN 145	PDAC	1.6	Alive	FOLFIRINOX + Radiation	Adjuvant chemo +radiotherapy
PIN 148	PDAC	1.8	Alive	None	No information
PIN 160	PDAC	1.4	Deceased	None	Gem
PIN 161	PDAC	-		-	
PIN 165	PDAC	-	Deceased	FOLFIRINOX +Short Course Radiation	No adj chemo
PIN 175	IPMN	-	Alive	None	Carbo/Etoposide
PIN 190	PDAC	-	Deceased	None	Gem/Abraxane *
PIN 191	PDAC	-	Alive	Gem/Oxaliplatin + Long Course Radiation	No information
PIN 194	PDAC	-	Deceased	Gem/Abraxane + Long Course Radiation	No information
PIN 199	PDAC	-	Alive	Neo-adjuvant—regime unknown	
PIN 205	PDAC	-	-	-	
PIN 210	NET	-	-	-	
PIN 211	PDAC	-	-	-	
PIN 212	MCN	-	-	-	
PIN 213	PDAC	-	-	-	
PIN 214	NET	-	-	-	
PIN 218	NET	-	-	-	
PIN 222	PDAC	-	-	-	
PIN 226	PDAC	-	-	-	
PIN 228	PDAC	-	-	-	
PIN 233	PDAC	-	-	-	
PIN 238	NET	-	-	-	
PIN 239	GIST	-	-	-	
PIN 266	Cholangio	-	-	-	
PIN 268	PDAC	-	-	-	
PIN 277	PDAC	-	-	-	
PIN 291	PDAC	-	-	-	

F/U = Follow-Up; Gem = Gemcitabine; * = (APACT study); “-“ information not available

**Table 3:** Tumour diagnosis, take rate and growth in first generation in mouse.

Sample Identifier	Diagnosis	Time to First Notice (days)	F1 Tumour Engraftment (%)	Tissue Used in Microarray Analysis
PIN 062	**ICC**	NT	0.0	-
PIN 065	**PDAC**	27.0	100.0	✓
PIN 080	**PDAC**	35.0	66.7	✓
PIN 082	**IPMN**	NT	0.0	-
PIN 089	**PDAC**	33.0	100.0	✓
PIN 091	**PDAC**	34.0	75.0	-
PIN 099	**PDAC**	29.0	25.0	-
PIN 102	Cholangio *	34.0	100.0	-
PIN 112	**PDAC**	196.0	33.3	✓
PIN 113	**PDAC**	34.0	100.0	-
PIN 115	**PDAC**	NT	0.0	-
PIN 116	**PDAC**	26.0	100.0	-
PIN 120	**PDAC**	56.0	100.0	✓
PIN 127	**PDAC**	62.0	100.0	-
PIN 132	**RCC**	NT	0.0	-
PIN 135	**NET**	NT	0.0	-
PIN 137	**PDAC**	NT	0.0	-
PIN 138	**PDAC**	NT	0.0	-
PIN 139	**PDAC**	98.0	33.3	-
PIN 140	**PDAC**	23.0	66.7	✓
PIN 141	**PDAC**	27.0	100.0	-
PIN 145	**PDAC**	NT	0.0	-
PIN 148	**PDAC**	NT	0.0	-
PIN 160	**PDAC**	27.0	100.0	✓
PIN 161	**PDAC**	42.0	66.7	✓
PIN 165	**PDAC**	NT	0.0	-
PIN 175	**IPMN/NET**	112.0	33.3	-
PIN 190	**PDAC**	306.0	33.3	-
PIN 191	**PDAC**	NT	0.0	-
PIN 194	**PDAC**	NT	0.0	-
PIN 199	**PDAC**	NT	0.0	-
PIN 205	**PDAC**	56.0	100.0	-
PIN 210	**NET**	NT	0.0	-
PIN 211	**PDAC**	NT	0.0	-
PIN 212	**Mucinous Cystic Neoplasm**	NT	0.0	-
PIN 213	**PDAC/Cholangio**	105.0	33.3	-
PIN 214	**NET**	NT	0.0	-
PIN 218	**NET**	20.0	66.7	-
PIN 222	**PDAC**	46.0	50.0	-
PIN 226	**PDAC**	NT	0.0	-
PIN 228	**PDAC**	NT	0.0	-
PIN 233	**PDAC**	78.0	33.3	-
PIN 238	**NET**	NT	0.0	-
PIN 239	**GIST**	NT	0.0	-
PIN 266	**Cholangio**	120.0	66.7	-
PIN 268	**PDAC**	118.0	33.3	-
PIN 277	**PDAC**	NT	-	-
PIN 291	**PDAC**	71.0	33.3	-

* Cholangio = Cholangiocarcinoma; “NT”= No tumour developed; “✓” = sample used in microarray study; “-“ = sample not used in microarray study.

**Table 4:** Breakdown of samples included in microarray profiling and matched comparisons.

Sample Identifier	PIN 065	PIN 080	PIN 089	PIN 112	PIN 120	PIN 140	PIN 160	PIN 161
Normal	**✓**	**✓**	**✕**	**✓**	**✓**	**✓**	**✓**	**✕**
Tumour	**✕**	**✓**	**✓**	**✓**	**✓**	**✓**	**✓**	**✓**
F1	**✓**	**✓**	**✓**	**✓**	**✓**	**✓**	**✓**	**✓**

“**✓**” = sample used in microarray study “**✕** “ = sample not used in microarray study

**Table 5:** The number of gene changes detected across the sample types, normal vs. tumour, tumour vs. F1 and triple comparison of normal vs. tumour vs. F1. All gene changes reported were statistically significant (*p* ≤ 0.05). Genes that were 2-fold or greater increased were investigated further.

Comparison	Criteria *p* ≤ 0.05	Number of Genes Changed
Normal vs. tumour	1.5-fold increase	1019
	2-fold increase	413
	1.5-fold decrease	394
	2-fold decrease	116
Tumour vs. F1	1.5-fold increase	1670
	2-fold increase	491
	1.5-fold decrease	2145
	2-fold decrease	1312
Normal vs. tumour vs. F1	1.5-fold increase	274
	**2-fold increase**	**89**
	1.5-fold decrease	94
	2-fold decrease	33
Normal vs. tumour vs. F1	1.5-fold increase in T (vs. N) and 1.5-fold decrease in F1 (vs. T)	147
	1.5-fold decrease in T (vs. N) and 1.5-fold increase in F1 (vs. T)	9

**Table 6:** Top 20 differentially expressed genes upregulated in a comparison of tumour vs. normal samples, ordered by fold change.

Gene Symbol	Tumour Avg (log2)	Normal Avg (log2)	Tumour SD	Normal SD	Fold Change	*p*-value
*SLC6A14*	7.6	3.1	1.9	1.9	22.8	4.00 × 10^−4^
*TSPAN1*	7.8	4.5	1.0	1.4	9.5	2.97 × 10^−5^
*TMPRSS4*	7.2	3.9	1.2	1.3	9.5	2.00 × 10^−4^
*MUC13*	8.9	5.6	1.6	1.7	9.4	7.00 × 10^−4^
*SLPI*	8.9	6.1	0.9	1.8	6.9	6.00 × 10^−4^
*SERPINB5*	5.3	2.5	0.9	1.0	6.8	8.84 × 10^−5^
*NQO1*	7.9	5.3	1.0	1.6	6.4	8.45 × 10^–5^
*HIST1H2BM*	6.2	3.5	1.3	1.7	6.4	6.10 × 10^–3^
*TOP2A*	6.4	3.7	0.7	1.4	6.4	2.00 × 10^–4^
*FERMT1*	7.6	4.9	1.1	0.9	6.4	3.00 × 10^–4^
*GALNT5*	6.5	4.0	1.7	1.2	5.8	3.89 × 10^–2^
*PLS1*	8.4	6.1	0.9	0.9	5.3	7.00 × 10^–4^
*TSPAN8*	8.0	5.6	0.6	1.2	5.2	3.00 × 10^–4^
*TMEM45B*	6.9	4.5	0.8	1.0	5.0	4.00 × 10^–4^
*AGR2*	5.7	3.4	1.4	1.3	5.0	1.50 × 10^–3^
*LIPH*	8.1	5.9	1.1	1.3	4.9	3.00 × 10^–4^
*KRT23*	7.5	5.3	0.6	1.3	4.5	8.80 × 10^–5^
*FAM83B*	6.8	4.7	1.2	1.3	4.5	5.00 × 10^–4^
*TPX2*	5.1	3.0	0.9	1.1	4.4	3.40 × 10^–3^
*C19orf33*	8.2	6.1	0.6	1.4	4.3	1.65 × 10^–2^

**Table 7:** Top 20 differentially expressed genes upregulated in a comparison of tumour vs. F1 samples, ordered by fold change.

Gene Symbol	PDX F1 Avg (log2)	Tumour Avg (log2)	F1 SD	Tumour SD	Fold Change	*p*-value
*SERPINB5*	8.0	5.3	1.0	0.9	6.6	8.96 × 10^–6^
*ABCA12*	6.3	3.7	1.1	1.0	5.7	2.34 × 10^–5^
*GALNT5*	9.0	6.5	2.1	1.7	5.6	4.14 × 10^–2^
*ASPM*	6.5	4.1	0.5	0.8	5.1	2.92 × 10^–7^
*AGR2*	8.1	5.7	0.6	1.4	5.1	3.00 × 10^–4^
*ANLN*	7.9	5.7	0.5	0.9	4.6	2.66 × 10^–5^
*AFAP1-AS1*	7.3	5.1	1.5	1.5	4.5	1.59 × 10^–2^
*TMEM45B*	8.9	6.9	1.0	0.8	4.1	1.10 × 10^–3^
*TPX2*	7.1	5.1	0.5	0.9	3.9	6.64 × 10^–5^
*CCNA2*	7.6	5.7	0.7	0.8	3.9	4.81 × 10^–5^
*TOP2A*	8.4	6.4	0.5	0.7	3.8	2.00 × 10^–4^
*FAM111B*	6.6	4.7	1.0	1.4	3.8	2.00 × 10^–4^
*S100A14*	8.7	6.8	0.8	0.9	3.8	5.70 × 10^–5^
*CDK1*	6.0	4.0	0.8	0.7	3.8	5.00 × 10^–4^
*FANCI*	6.7	4.8	0.6	0.9	3.7	2.36 × 10^–5^
*FUT2*	7.0	5.1	1.0	0.7	3.6	6.00 × 10^–4^
*HIST1H2BM*	8.1	6.2	0.7	1.3	3.6	2.50 × 10^–2^
*CENPF*	6.8	4.9	0.3	0.6	3.6	3.70 × 10^–7^
*KIF11*	6.8	5.0	0.6	0.8	3.5	2.87 × 10^–5^
*MKI67*	7.7	5.91	0.49	0.79	3.5	1.00 × 10^–4^

**Table 8:** Gene ontology analysis of shortlisted genes by biological process.

GO Biological Process Complete	Homo Sapiens—REF LIST (20,996)	Query List (89)
cell cycle (GO:0007049)	1328	21
cell cycle process (GO:0022402)	986	18
regulation of cell cycle (GO:0051726)	1206	17
mitotic cell cycle process (GO:1903047)	610	16
mitotic cell cycle (GO:0000278)	696	16
cell division (GO:0051301)	494	15
regulation of cell cycle process (GO:0010564)	763	13
regulation of mitotic cell cycle (GO:0007346)	638	12
positive regulation of cellular protein localization (GO:1903829)	332	9
regulation of mitotic nuclear division (GO:0007088)	171	8
regulation of nuclear division (GO:0051783)	196	8
nuclear division (GO:0000280)	279	8
DNA packaging (GO:0006323)	177	7
chromosome condensation (GO:0030261)	43	5

**Table 9:** Amigo database analysis of shortlisted genes by biological process.

GO Biological Process Complete	Homo Sapiens—REF LIST (20,996)	Upload List (89)	Upload (Fold Enrichment)	Upload (*p*-Value)
cell cycle (GO:0007049)	1335	21	3.71	1.39 × 10^–3^
cell cycle process (GO:0022402)	998	18	4.25	1.80 × 10^–3^
mitotic cell cycle process (GO:1903047)	616	16	6.13	7.34 × 10^–5^
mitotic cell cycle (GO:0000278)	699	16	5.4	4.10 × 10^–4^
cell division (GO:0051301)	496	15	7.13	3.15 × 10^–5^
regulation of mitotic nuclear division (GO:0007088)	173	8	10.91	8.88 × 10^–3^
regulation of nuclear division (GO:0051783)	197	8	9.58	2.26 × 10^–2^
chromosome condensation (GO:0030261)	43	5	27.43	1.60 × 10^–2^
Unclassified (UNCLASSIFIED)	3111	4	0.3	

## Supplementary Materials

Supplementary Materials can be found at https://www.mdpi.com/1422-0067/21/3/962/s1; Figure S1: Representative H&E images of patient tumours in comparison to corresponding PDX F1 tumours of PIN 065 (A & B), PIN 080 (C & D), PIN 089 (E & F) PIN 091 (G & H), PIN 099 (I & J); Figure S2: Mutations detected by Sequenom MassArray Analysis in 10 PDX F1 tumour samples; Table S1: Gene and mutations detected in 10 F1 PDX samples by Sequenom MassArray Analysis; Table S2: Shortlist of selected 89 genes increased in T compared to N and also in F1 compared to T; Table S3: List of mutations analysed using the Agena MassArray technology.
