# Supplementary material for: Establishment and Characterisation by Expression Microarray of Patient-Derived Xenograft Panel of Human Pancreatic Adenocarcinoma Patients
Source: Int J Mol Sci. 2020 Jan 31;21(3):962. doi: 10.3390/ijms21030962 (PMC7037178; doi:10.3390/ijms21030962)
Supplement: Supplementary file 1 [file ijms-21-00962-s001.zip › Supp Fig S1&S2.pdf]

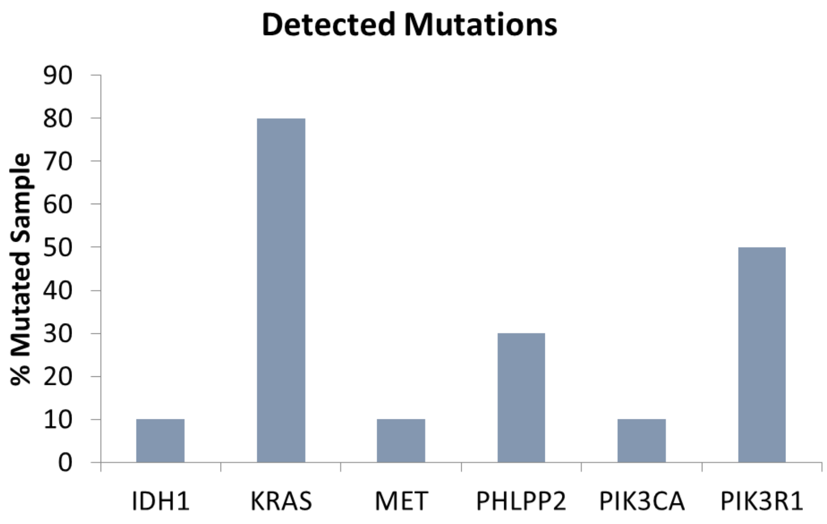

Figure S1: Mutations detected by Sequenom MassArray Analysis in 10 PDX F1 tumour samples.

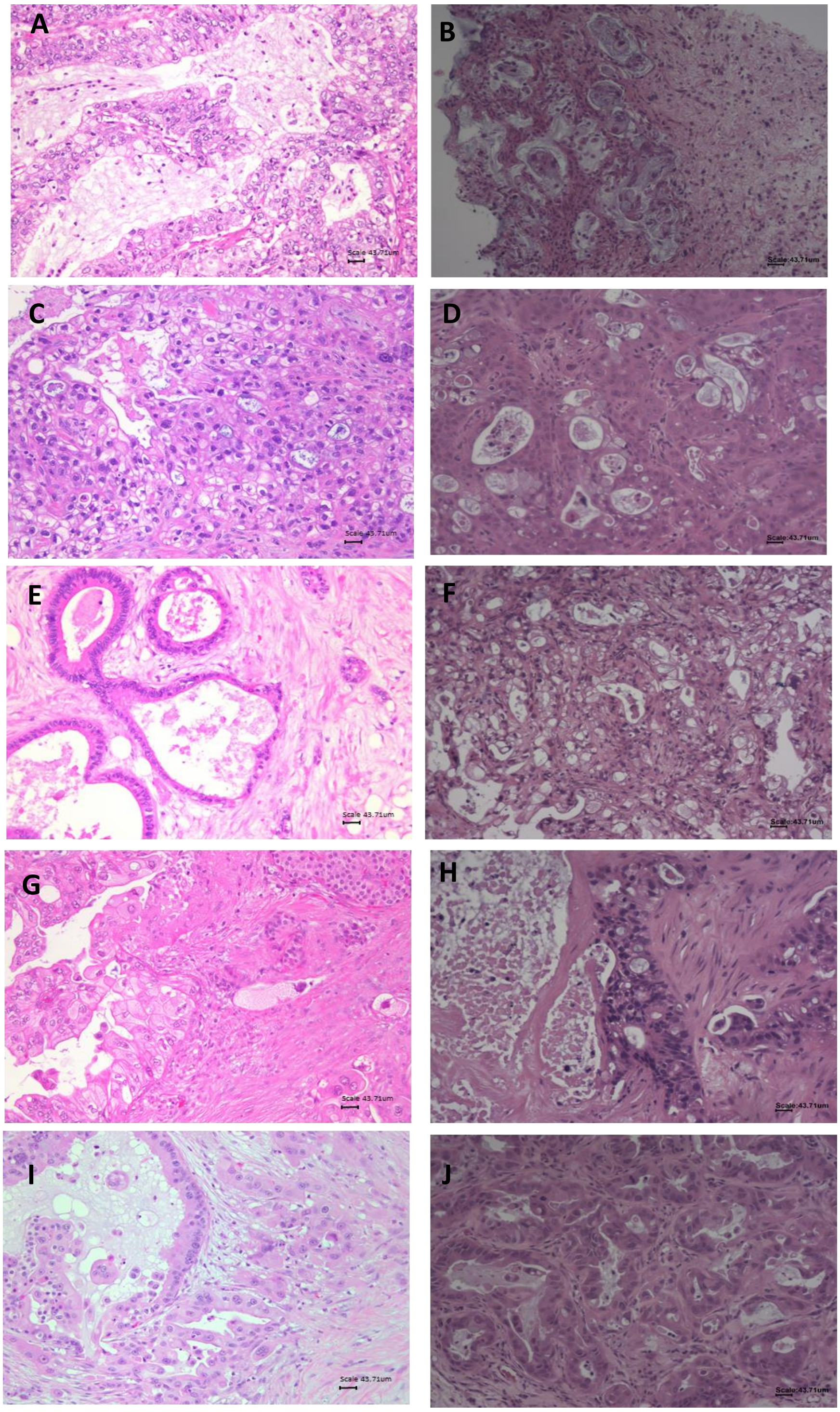

Figure S2: Representative H&E images of patient tumours in comparison to corresponding PDX F1 tumours of PIN 065 (A & B), PIN 080 (C & D), PIN 089 (E & F) PIN 091 (G & H), PIN 099 (I & J). Images A, C, E, G and I are patient tumour images and images B, D, F, H, J are PDX tumour images. Magnification for all images is 20x
