# Supplementary material for: Establishment and Characterisation by Expression Microarray of Patient-Derived Xenograft Panel of Human Pancreatic Adenocarcinoma Patients
Source: Int J Mol Sci. 2020 Jan 31;21(3):962. doi: 10.3390/ijms21030962 (PMC7037178; doi:10.3390/ijms21030962)
Supplement: Supplementary file 1 [file ijms-21-00962-s001.zip › Supplementary Table S2.docx]

Supplementary Table S2: List of mutations analysed using the Agena MassArray technology

| **AKT1** | E17K, E49K, G173R, K179M |
| --- | --- |
| **AKT2** | E17K, G175R |
| **AKT3** | E17K, G171R |
| **ALK** | L560F, A877S, D1091N, M1166R, I1171N, F1174C/S/L/L/I/V, F1245C/L/V/I, R1275Q/L |
| **APC** | R1114X, E1306X, E1338X, Q1367X, E1379X, Q1429X, R1450X |
| **BRAF** | R444Q, R462I, I463S, G464E/V/A/R, G466R/E/V/A, G469A/E/R/V, V471F, Y472S, E586K, D587A/E, I592M/V, D594E/V/G, F595L/L/L/S, G596R, L597R/R/Q/V, T599I, V600E/A/G/L/M, K601E/N/N, S605N, G615R |
| **CDK4** | R24C/H |
| **CDKN1B** | P117S |
| **CDKN2A** | R58X, E61X, E69X, R80X, H83Y, E88X |
| **CTNNB1** | A13T, A21T, V22A, D32A/G/V/H/N/Y, S33A/P/T, G34E/V/A/R/R, I35N/S/T, H36P/R/Y, S37A/P/T/C/F/Y, T41A/P/S/I/N/S, S45A/P/T/C/F/Y |
| **DDR2** | R105S, N456S, T533K |
| **EGFR** | V689M, N700D, E709A/V/G/K/Q, G719A/D/C/S/R, S720T/P, D761N/Y, V769L/M, T783A, A839T, K846R, L858M/R, L861Q/R, G863D, H870R, E844K |
| **ERBB2** | S310F/Y, L755S, G776S/V, D769H, V777A/L/M, V842I, H878Y |
| **FBXO4** | S8R, S12L, L23Q, P76T |
| **FBXW7** | R465C/H/L, R479G/Q/L, R505C/S/H/L/P, S582L |
| **FGFR1** | S125L, P252T |
| **FGFR2** | S252W, Y375C, N549K/K |
| **FGFR3** | R248C, S249C, G370C, S371C, Y373C, G380R, A391E, K650E/Q/M/T, G697C |
| **GNA11** | Q209L/P, R138C |
| **GNAS** | R201H/S/C, Q227H/L/R |
| **GNAQ** | Q209L/P/R |
| **HRAS** | G12S/R/C/D/A/V, G13S/R/C, Q61H/H/Q/K/L/P/R, E62G |
| **IDH1** | G70D, R132C/G/S/H/L, V178I |
| **IDH2** | R172G/W/M/K/S |
| **KIT** | M552L, Y553N, W557G/R/R, K558N/R, V559A/D/G/I, V560D/A, G565R, N566D, Y568D, V569G, P573L, F584S, L576P, E561K, K642E, V654A, T670I, D716N, D816E/H/N/Y/G/V/A, D820E/E/H/Y/A/G, N822K/N/K/Y/H, Y823D/N |
| **KRAS** | G12D/A/V/S/R/C, KRASG13D/A/V/S/R/C, L19F/F, Q22K, T58I, A59T/G/E, G60D, Q61E/K/X/H/H/Q/L/P/R, A146P/T |
| **MAP2K1** | F53C/S, Q56P, K57N, P124L/T/S, E203K/Q |
| **MAP2K2** | E207K/Q, R388Q |
| **MAP3K13** | P373S, S694L, R880C, A882S |
| **MET** | E168D, N375S, R970C, T1010I, R1112R/L/Y, H1124D, M1131T, Y1248C/H/D, Y1253D, M1268T |
| **MLH1** | V384D |
| **MYC** | P57S, T58A |
| **NCOR1** | R108X, Q313X, E379X, I1422S, Q1792X |
| **NRAS** | G12D/A/V/S/R/C, G13D/A/V/S/R/C, A18T, Q61E/K/X/H/H/Q/R/P/L |
| **PDGFRA** | V561D, N659K/Y, D842Y/N/V, D846Y, Y849C, D1071N |
| **PIK3CA** | R38H, Q60K, R88Q, K111N, G118D, N345K, S405F, E418K, C420R, E453K, P539R, E542K/Q/V/G, E545D/K/Q/A/V/G, Q546H/E/K/L/P/R, C901F, F909L/L, M1004I, G1007R, Y1021C/H/N, R1023Q, T1025A/S/I, A1035T/V, M1043I/I/I/V, A1046V, H1047R/L/Y, G1049R, I1058F, H1065L, |
| **PIK3R1** | G376R, D560Y, N564D |
| **PTEN** | R130L/P/Q/X, R173C/H, R233X, R335X |
| **PTPN11** | S72D/V/T, E69K, E76A/G/V/Q/K |
| **RB1** | E137X, L199X, R320X, R358X, R455X, R552X, R556X, R579X, C706F, E748X |
| **RET** | C634R/W/Y, A664D, E768D, M918T |
| **SMARCD** | Q539X, D391H, Q504X |
| **SOS1** | R248H, R688Q, H888Q |
| **SRC** | Q531X |
| **STK11** | Q37X, Q170X, D194N/Y/V, G196V, E199X/K, P281L, W332X, F354L |
| **TBX3** | Y163X, W197X |
| **VHL** | P81X, L85P, L89H, L158Q/V, R161X, R167W |
